# Major depressive disorder after heart transplantation with partial response to antidepressant therapy and remission with aripiprazole augmentation: A case report

**DOI:** 10.1002/npr2.12463

**Published:** 2024-06-25

**Authors:** Toshinori Nakamura, Yuta Kuraishi, Dai Ohya, Kazuhiro Kimura, Daimei Sasayama, Shinsuke Washizuka

**Affiliations:** ^1^ Department of Psychiatry Shinshu University School of Medicine Matsumoto Nagano Japan; ^2^ Department of Cardiovascular Medicine Shinshu University School of Medicine Matsumoto Nagano Japan

**Keywords:** antidepressant, antipsychotics, aripiprazole, depression, heart transplant

## Abstract

The incidence of major depressive disorder (MDD) after heart transplantation is high; however, there are no reports on treatment options when antidepressant therapy fails to improve the condition. We herein report on the case of a woman with MDD after heart transplantation who partially improved with antidepressant treatment but continued to have a loss of appetite. Augmentation treatment with aripiprazole improved her appetite, and her MDD went into remission. When antidepressant treatment is not sufficiently effective for MDD after heart transplantation, augmentation treatment with antipsychotics, such as aripiprazole, should be considered.

## Abstract 2

A woman diagnosed with major depressive disorder (MDD) following a heart transplant experienced partial improvement with antidepressant treatment but continued to suffer from loss of appetite. Augmentation treatment with aripiprazole improved her appetite, and her MDD went into remission. When antidepressant treatment alone is not sufficiently effective for post‐heart transplantation MDD, augmentation treatment with antipsychotics, such as aripiprazole, should be considered.

## Graphical abstract

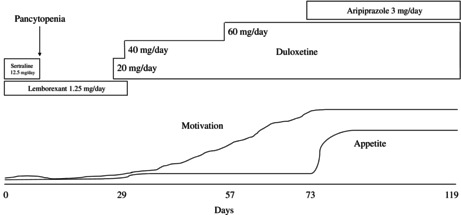

## 1 INTRODUCTION

The incidence of major depressive disorder (MDD) after heart transplantation is high, with a reported rate of 26.3% within 1 year after transplantation.^1^ Patients who develop MDD after heart transplantation have a lower 5‐year survival rate; However, patient with MDD after heart transplantation, who receive treatment for MDD, have survival rate similar to those without MDD.^2^ Therefore, treatment of MDD is important.^1^, ^3^ Augmentation of antidepressant therapy with aripiprazole is effective in the treatment of MDD and is commonly used in psychiatric settings, although it is associated with akathisia as an adverse effect.^4^, ^5^ We herein report a case of a patient with depressive episodes after heart transplantation treated with aripiprazole after an insufficient response to antidepressants.

## 2 CASE PRESENTATION

The present case involved a 61‐year‐old woman with no history of psychiatric condition among blood relatives. She had no history of alcohol consumption, smoking, or illegal drug use. In July, at the age of 55 years, she was enrolled for heart transplantation due to dilated cardiomyopathy due to Becker's type muscular dystrophy carrier with deletion of exons 63–74. After hospitalization and implantation of a left ventricular assist device, the patient presented with sleep disturbances, anxiety, and agitation, which necessitated psychiatric intervention. We determined that the anxiety was due to the patient's physical illness and not MDD and stabilized once the patient understood the current situation and the treatment process until discharge. Medication consisted of anxiolytics and hypnotics as needed, and the psychiatric intervention was terminated in November. At the age of 58 years, she experienced a cerebral infarction in the left middle cerebral artery region, which left her with motor aphasia, dysarthria, and right hemiplegia. No anxiety or depressive episodes were observed after the stroke. Her heart transplantation was performed at another hospital in December at 60 years of age. The patient had a good postoperative course with minimal rejection. However, because of the after‐effects of the cerebral infarction, rehabilitation did not progress, and proper food intake suffered. She was transferred to our hospital in January of the following year, where she had been undergoing continuous treatment since before the surgery. After the transfer, her appetite and motivation for rehabilitation temporarily improved; however, her energy decreased, and she could no longer proceed with rehabilitation or consume food. Based on her declining motivation and appetite and her dark facial expression, MDD was suspected, and a psychiatric intervention was performed in March at the age of 61 years.

The patient had not consulted a psychiatrist for 5.5 years, but she recognized the doctor who had examined her by his voice during the interview on the 1st day of the psychiatric intervention. However, there was no change in her facial expression, and she did not attempt to face the interviewers while lying in bed. Although she could answer the questions appropriately, she appeared exhausted and had difficulty concentrating. Interest in rehabilitation and other activities was significantly reduced. Her appetite was severely decreased, and she could hardly eat or drink; consequently, her weight decreased rapidly, and dehydration was observed. Therefore, intravenous hyperalimentation was started on the 1st day, and the examination was discontinued shortly. Based on the Diagnostic and Statistical Manual of Mental Disorders, Fifth Edition,^6^ MDD was diagnosed, and sertraline was initiated on the 2nd day of intervention. Despite the low initial dose of 12.5 mg/day, she soon developed pancytopenia; therefore, sertraline was discontinued on the 9th day of intervention.

**FIGURE 1:**
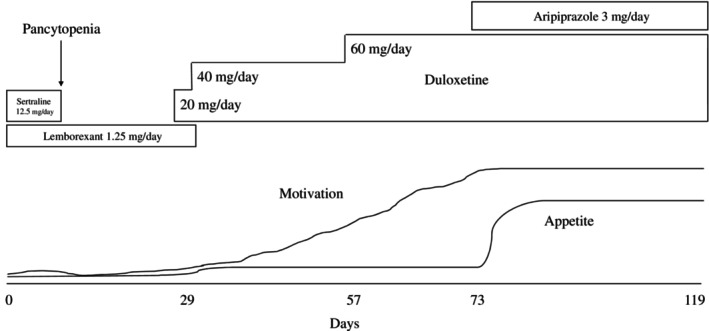
Motivation, appetite, and neuropsychotropic during psychiatric intervention.

After the improvement of the pancytopenia and a short stay at the hospital where the heart transplantation was performed, the patient continued to have depressive episodes. Therefore, duloxetine (20 mg/day) was started on the 29th day of the intervention. The dose of duloxetine was increased to 40 mg/day on the 36th day after it was confirmed that there were no side effects. After approximately 1 week, the patient's facial expression softened, and she began to smile. As the patient's motivation continued to decrease, the dose was increased to 60 mg/day on the 57th day. Thereafter, the patient's motivation for rehabilitation improved. However, as the patient's appetite continued to decrease, aripiprazole (3 mg/day) was started on the 73rd day of intervention to augment the antidepressant effect. After starting aripiprazole, her appetite gradually improved; she was able to eat and no longer required intravenous hyperalimentation on the 87th day. The patient was discharged from the hospital 119 days after the intervention (Figure 1). Her food intake did not decrease after discharge. Through the course of treatment, the prednisolone dose was reduced from 5 mg/day to 2 mg/day. The immunosuppressive agents, mycophenolate mofetil and tacrolimus hydrate, were adjusted from 1000 to 1500 mg/day and 0.5 to 2 mg/day, respectively.

## 3 DISCUSSION

This is the first report of augmentation therapy with aripiprazole in addition to antidepressant therapy in patients with MDD after heart transplantation. After heart transplantation, the patient presented with depressive episodes, consisting mainly of loss of motivation and appetite. The patient had motor aphasia due to the after‐effects of cerebral infarction, and it was difficult for her to verbalize her depressive episodes, making it difficult to conduct clinical rating scales; therefore, we diagnosed MDD based on her behavior, such as motivation and appetite. Motivation improved with duloxetine, but appetite remained depressed. Therefore, aripiprazole was added and the appetite improved. New‐generation antidepressants, such as selective serotonin reuptake inhibitors, are the first choice for the treatment of MDD after heart transplantation.^7^ In the standard treatment of psychiatric patients with MDD, aripiprazole is often used as an augmentation therapy when antidepressants are only partially responsive. Antipsychotics potentially cause drug‐induced long QT syndrome and risk of sudden death due to arrhythmias.^8^ However, serum concentrations of antipsychotics within the therapeutic range have a rather small contribution to QTc prolongation compared to genetic factors and age of individual patients^9^; Moreover, aripiprazole does not cause QTc prolongation in older adults or patients with increased cardiometabolic problems^10^; therefore, it was easy to administer and well‐tolerated in this patient with heart transplantation. Prednisolone is known to increase the incidence of depression^11^; however, in this case, the low dosage did not seem to affect the disease course considerably.

## 4 CONCLUSION

Antidepressant treatment for depressive episodes after heart transplantation should be administered at sufficient doses for appropriate periods. If ineffective, augmentation therapy with aripiprazole may be considered in the treatment of MDD that develops under specific conditions, such as that after heart transplantation.

## AUTHOR CONTRIBUTIONS

TN, YK, DO, and KK were involved in the clinical investigation. TN wrote the first draft of the manuscript. TN, DS, and SW edited the manuscript. All authors read and approved the final manuscript.

## FUNDING INFORMATION

This research did not receive any specific grant from funding agencies in the public, commercial, or not‐for‐profit sectors.

## CONFLICT OF INTEREST STATEMENT

TN has received honoraria for lectures from Otsuka Pharmaceutical, Eisai, Sumitomo Pharma, MSD, Janssen Pharmaceutical K, Lundbeck Japan, and Takeda Pharmaceutical. KK has received honoraria for lectures from AstraZeneca, Ono Pharmaceutical, Kowa, Nippon Shinyaku, Jansen Pharmaceutical, Mochida Pharmaceutical, Boehringer Ingelheim, and Daiichi‐Sankyo. DS has received honoraria for lectures from Otsuka Pharmaceutical, Takeda Pharmaceutical, and Shionogi Pharma Co., Ltd. SW has received research grants from Japan Society for the Promotion of Science, Otuka Pharmaceutical, Eisai, Pfizer, Daiichi Sankyo, Tsumura, Mochida, Astellas, Shionogi, Takeda Pharmaceutical, and Sumitomo and honoraria for lectures from Otuka Pharmaceutical, Eli Lilly, MSD, Yoshitomiyakuhin, Daiichi Sankyo, Sumitomo, Eisai, Kyowa, Janssen, Pfizer, Takeda Pharmaceutical, Viatris, and Towa.

## ETHICS STATEMENT

Approval of the research protocol by an Institutional Reviewer Board: N/A.

Informed Consent: An explanation was given to the patient that the case report would be made in such a way that the individual could not be identified, and the consent of the individual was obtained in writing.

Registry and the Registration No. of the study: N/A.

Animal Studies: N/A.

## Data Availability

Data sharing is not applicable to this article as no datasets were generated or analyzed during the current study.
